# Automated comparison of last hospital main diagnosis and underlying cause of death ICD10 codes, France, 2008–2009

**DOI:** 10.1186/1472-6947-14-44

**Published:** 2014-06-05

**Authors:** Agathe Lamarche-Vadel, Gérard Pavillon, Albertine Aouba, Lars Age Johansson, Laurence Meyer, Eric Jougla, Grégoire Rey

**Affiliations:** 1 Inserm, CépiDc (Epidemiology center on medical causes of death), CHU Bicêtre, 80 rue du Général Leclerc, Kremlin Bicêtre, CEDEX 94270, France; 2 Inserm, UMRS 1018, Kremlin-Bicêtre, France; 3 Université Paris Sud, Kremlin-Bicêtre, France; 4 Swedish National Board of Health and Welfare, Center for Epidemiology, Stockholm, Sweden; 5 AP-HP, CHU Bicêtre, Service de Santé Publique et d’Epidémiologie, Kremlin-Bicêtre, France

**Keywords:** Cause of death, Death certificate, Medical coding, Hospital mortality, Quality indicators, Health care, Medical record linkage

## Abstract

**Background:**

In the age of big data in healthcare, automated comparison of medical diagnoses in large scale databases is a key issue. Our objectives were: 1) to formally define and identify cases of independence between last hospitalization main diagnosis (MD) and death registry underlying cause of death (UCD) for deceased subjects hospitalized in their last year of life; 2) to study their distribution according to socio-demographic and medico-administrative variables; 3) to discuss the interest of this method in the specific context of hospital quality of care assessment.

**Methods:**

1) Elaboration of an algorithm comparing MD and UCD, relying on Iris, a coding system based on international standards. 2) Application to 421,460 beneficiaries of the general health insurance regime (which covers 70% of French population) hospitalized and deceased in 2008–2009.

**Results:**

1) Independence, was defined as MD and UCD belonging to different trains of events leading to death 2) Among the deaths analyzed automatically (91.7%), 8.5% of in-hospital deaths and 19.5% of out-of-hospital deaths were classified as independent. Independence was more frequent in elder patients, as well as when the discharge-death time interval grew (14.3% when death occurred within 30 days after discharge and 27.7% within 6 to 12 months) and for UCDs other than neoplasms.

**Conclusion:**

Our algorithm can identify cases where death can be considered independent from the pathology treated in hospital. Excluding these deaths from the ones allocated to the hospitalization process could contribute to improve post-hospital mortality indicators. More generally, this method has the potential of being developed and used for other diagnoses comparisons across time periods or databases.

## Background

Because of their richness, availability and marginal cost, medico-administrative data are increasingly used for epidemiological studies and health care performance assessment [1]. The linkage of different databases gives even more possibilities to address important public health questions. However, the mere juxtaposition of information may be insufficient and the data sometimes need to be studied in relation with each other. In particular, the relationship between medical diagnoses recorded at different times or in different contexts for an individual may be of interest. For example, the study of the frequency and causes of death after medical care may be very informative, whether focalized on a specific care and specific subsequent mortality causes [2], or in more general approaches like quality of hospital care assessment. Along this line, there is a growing interest in assessing the contribution of the causes of death information to the building of post-hospital mortality indicators [3]. However, comparing two medical diagnoses in order to assess whether they refer to a unique morbid process (or in other words whether they are consistent), or not, is complex. Moreover, given the huge size of national databases, this complex comparison needs to be performed automatically.

Hospital diagnoses have been compared to the underlying causes of death in a few studies on death certification quality assessment [4-8]. In this context, Johansson and Westerling have proposed in 2002 a method of comparison of ICD9 codes [7]. This method takes advantage of the validated automatic systems developed for the selection of the underlying cause of death. These systems, following very precise international definitions and procedures, are designed to check the chain of events leading to death by testing causal relations between medical conditions. Iris [9,10], a validated piece of software which relies on the current international standards of death certification [11], makes the update and an extension of this method possible. Our purpose was to test the feasibility of using such a system in order to compare individual diagnoses derived from the French hospital and causes of death databases.

The aim of this study was 1) to propose and test a reproducible, automatic method of comparison of the main diagnosis of last hospital stay to the underlying cause of death in order to determine their consistency or independence 2) to study the distribution of consistency and independence according to socio-demographic and medico-administrative variables for deceased subjects hospitalized at least once during their last year of life.

## Methods

### Data sources

#### Cause-of-death data

The French death certificates are complying with the WHO international standards. They are exhaustively collected by the Epidemiological Center for the Medical Causes of Death (Inserm -CépiDc) [12]. Since 2000, causes of death are coded according to the 10th revision of the International Classification of Diseases (ICD-10) [11]. This analysis includes all the causes mentioned on the death certificate, 3.4 on average, plus the UCD determined by the ICD-10 rules. The UCD can be one of the causes mentioned on the death certificate or a combination of these causes in a single code (e.g. Diabetes with renal complication).

#### Hospitalization data

The French acute care hospital database (PMSI-MCO) [13] is designed for hospital payment. It provides medical information for all patients discharged from short-stay hospitals, both public and private. Patient's stays are chained so that the number of hospitalizations within the year before death can be calculated. This study focuses on the last hospitalization before death (both occurring in 2008 or 2009). The patient's gender, age (at admission), and the main diagnosis (ICD10 code) were extracted. The hospitalization database is included in the Social Security database (SNIIRAM) [14].

#### Linkage

By the time of the analysis, vital status and date of death of the deceased were available only for the beneficiaries of the general health insurance regime. This population accounts for about 70% of French residents (it does not include state employees, students, self-employed, agricultural workers and farmers). Among those hospitalized during the year preceding their death, 96.4% of these beneficiaries could be linked to a single death certificate. The matching was performed through a deterministic methodology allowing at most one difference on one of the following indirect patient identifiers: year and month of birth; year (this variable had to match), month, and day of death; gender; *département* and *commune* of residence. Only unique matches were kept in the final set.

Infants deceased before one year of age were excluded because the quality of the vital status assessment for this age-class could not be precisely known. Besides, since the discharge-death time interval was imprecise for 2008, the 2008 records were considered only when death occurred in hospital, or 3 months or more after discharge (exact day of discharge available in 2009, month of discharge only in 2008).

The final database comprises 421,460 subjects deceased in the year following their last discharge.

The linkage of the hospitalization and cause of death data and the study of the resulting dataset were approved by the two French data protection committee and institutional ethical review boards concerned: Institut des Données de Santé (authorization n°16-24/11/2010) and Commission Nationale de l'Informatique et des Libertés (authorization n° 1454315).

### Definitions

The French definition of the main diagnosis has changed during the study period, from "condition that takes up the majority of resource use during the hospital stay" before march 2009, to "final diagnosis explaining hospital admission" after. However, this modification of definition had no impact on our results (results not shown).

In order to capture the pathology, which is the relevant information in our purpose, when the *main diagnosis* of the hospital database was a chapter XXI code (Factors influencing health status and contact with health services), the "main diagnosis" (MD) mentioned in this article was defined as the *related diagnosis*.

UCD is defined in volume 2 of ICD-10th revision as "(a) the disease or injury which initiated the train of morbid events leading directly to death, or (b) the circumstances of the accident or violence which produced the fatal injury".

### Comparison method

#### Classification

The aim was to compare MD and UCD in order to analyze their independence or consistency.

Consistency was defined as MD and UCD belonging to a same train of events leading to death. If the quality of the information held in both codes seemed sufficient and if MD and UCD could not belong to a same train of events leading to death, they were considered independent.

Four cases were distinguished:

– Similarity: MD and UCD refer to the same morbid condition, even if precision levels may differ (Eg1: UCD = Intracerebral haemorrahage, unspecified (I61.9) and MD = Intracerebral haemorrahage in cortical hemisphere (I61.1). Eg2: UCD = Pneumonia, unspecified (J18.9) and MD = Bacterial pneumonia, unspecified (J15.9)).

– Acceptable sequence: the two codes refer to different conditions but belong to a same train of events leading to death. Because UCD is defined as the cause that initiated the process, UCD can precede MD in the causal sequence, even though death occurs chronologically at the end of or after the last hospital stay. Acceptable sequences correspond to cases where MD is a complication of UCD (Eg1: UCD = Alcoholic cirrhosis of liver (K70.3) and MD = Rupture of esophageal varices (I85.0) . Eg2: UCD = Malignant neoplasm of bronchus and lung, unspecified (C34.9) and MD = Secondary malignant neoplasm of brain and cerebral meninges (C79.3)).

– Independence: both conditions belong to different trains of events leading to death (Eg: UCD = Calculus of bile duct with cholangitis (K80.3) and MD = Primary coxarthrosis, bilateral (M16.0)).

– Non-informative death certificate: cases that cannot be interpreted in terms of similarity, acceptable sequence or independence because UCD is not informative (Eg: UCD = Cardiac arrest, unspecified (I46.9)).

Similarities and acceptable sequences compose the consistent cases.

#### Algorithm

In order to classify each death in one of these four cases, an algorithm was designed to compare MD of last stay and UCD, taking all medical conditions mentioned on the death certificate into account (see Additional file 1).

At four stages of the algorithm, the type of relationship between MD and UCD was given by running Iris software (V. 4.0.38) on "test certificates" (see Additional file 2). Iris is a language-independent coding system using international standards [9,10] (see Additional file 3): the WHO ICD-10 classification, rules and guidelines as well as the knowledge base of the Mortality Medical Data System (MMDS) [15,16], ACME (Automatic Classification of Medical Entry) software in particular [17,18].

Artificially introducing MD in a test certificate, at a specific place according to the question asked, permitted us to assess its potential participation in the causal sequence leading to death (5). This method is an update and an extension of the one first proposed by Johansson and Westerling [7].

### Statistical analysis methods

In-hospital and out-of-hospital deaths were analyzed separately. The relationships between MD and UCD were studied according to age, gender, discharge-death time interval (in months) and main ICD Eurostat Shortlist chapters of UCD (corresponding ICD chapters): neoplasms (Chapter II), diseases of the nervous system and the sense organs (Chapters VI & VII), circulatory (Chapter IX), respiratory (Chapter X), and digestive (Chapter XI) systems, external causes of morbidity and mortality (Chapter XX), and one class for Others. Because they do not hold any information about the organs originally implied in the death process, imprecise UCDs (ICD-10 code in R99, R96.0, R57.9, R40.2, R09.2, I46.9, I99, I95.9, J96.0, J96.9, P28.5) were excluded from the comparison according to the category of UCD.

Univariate and multivariate log-binomial regression analysis [17] were used to study risk factors for independence vs. consistency, excluding non-informative cases. Relative risks (RRs) of independence and their 95% confidence intervals were estimated, crude and adjusted for age, gender, discharge-death time interval, length of stay, number of stays during the last year of life, and category of UCD. For each variable, the modal class was chosen as reference class.

Age, discharge-death time interval, length of stay, and number of stays during last year of life were included as continuous variables in order to perform trend tests.

Analyses were performed with SAS® version 9.3.

## Results

The study population comprised 323,375 subjects deceased in-hospital and 98,085 deceased out of hospital.

The automatic method relying on Iris software was able to classify the relationship between MD and UCD for 91.7% of this population. The main reasons for rejects by Iris were MD not accepted as valid causes of death (Chapter XXI codes) and diagnoses implying iatrogenicity (which have to be handled manually).

### Place of death in/out hospital

MD and UCD were consistent in 88.8% of in-hospital deaths and in 72.9% of the deaths occurring out of hospital (Table 1).

**Table 1:** Relationship between main diagnosis (MD) and underlying cause of death (UCD) according to the place of death in/out hospital

**Relationship UCD/MD**	**In-hospital deaths % (n = 298 083)**	**Out-of-hospital deaths % (n = 88 403)**
**Similarity (a)**	40.2	23.1
**Acceptable sequence (b)**	48.6	49.8
***Subtotal Consistency (a + b)***	***88.8***	***72.9***
**Independence**	8.5	19.5
**Non-informative UCD**	2.7	7.6
**Total**	**100.0**	**100.0**

Both independencies and non-informative UCD were higher for out-of-hospital than for in-hospital deaths: 19.5% vs. 8.5% and 7.6% vs.2.7% respectively.

### Age and gender

On the whole, independence increases with age. However, considering the 15–34 age class, the proportion of independence was the lowest of all age classes for in-hospital deaths, and the highest for out-of-hospital deaths (results not shown).

UCDs are more often non-informative for deaths of age class 85 years and over than below 85 years: 4.8% vs. 2.1% in-hospital and 10.2% vs. 6.0% out-of-hospital. Non-informative UCDs were higher in females but this result was largely attenuated after adjustment for age.

### Discharge-death time interval

**Figure 1:**
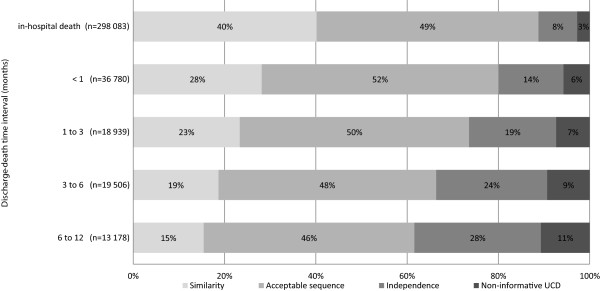
Relationship between main diagnosis (MD) and underlying cause of death (UCD) according to the discharge-death time interval (in months).

Whereas the proportion of acceptable sequences remained roughly constant around 49%, similarities decreased progressively from 40.2% for in hospital deaths to 15.4% when death occurred more than 6 months after discharge, resulting in a decrease of consistency (acceptable sequence + similarity) from 88.8% to 61.6% (Figure 1).

As the discharge-death time interval increases, the proportions of both independencies and non-informative UCD increased. MD and UCD were independent in 8.5% of in-hospital deaths and 27.7% of out-of-hospital deaths occurring between six months and one year after discharge. The proportion of non-informative UCD reached 10.7% for deaths happening between six months and one year after discharge.

### Cause of death

**Figure 2:**
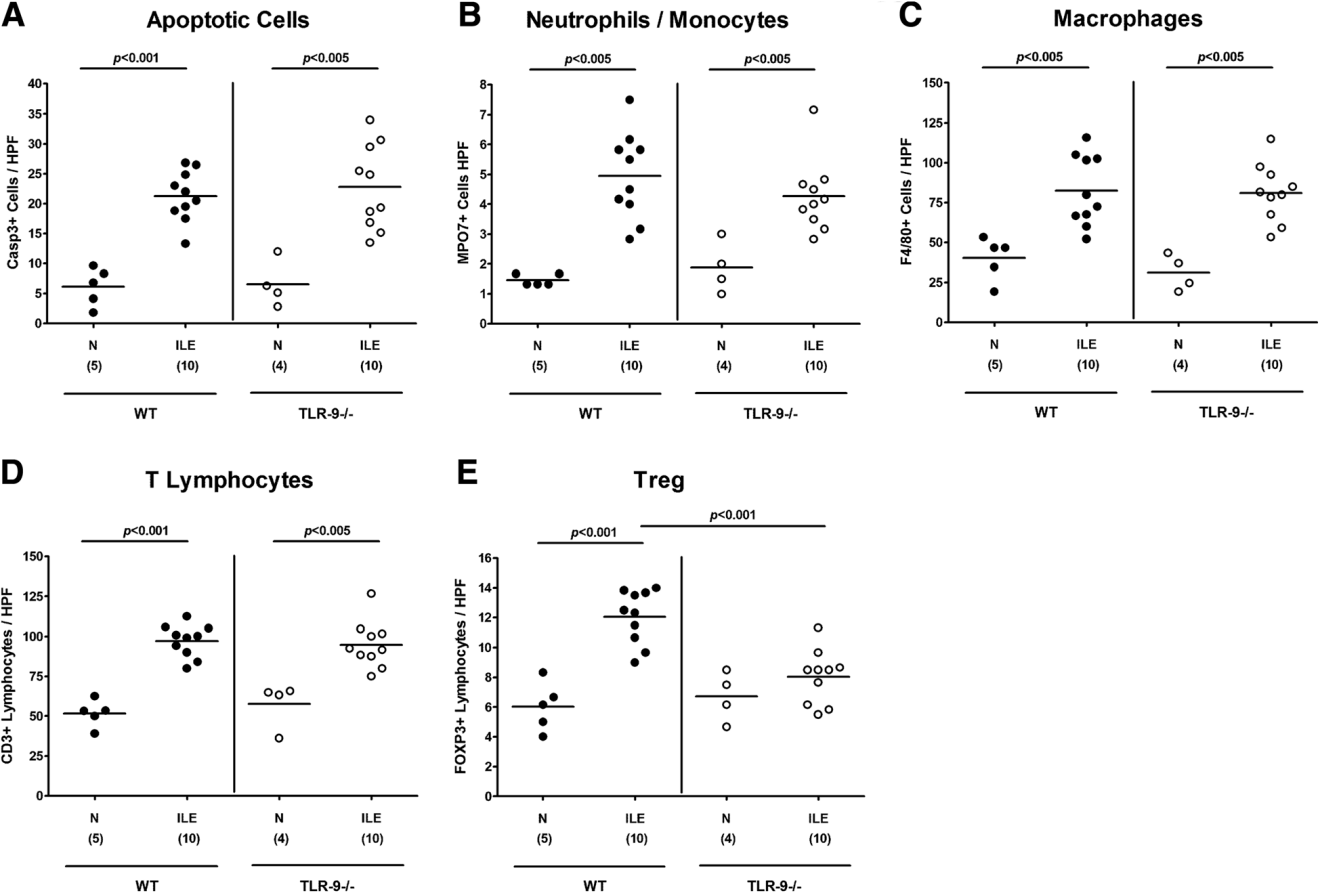
**Relationship between main diagnosis (MD) and underlying cause of death (UCD) according to the main ICD chapter of UCD.** Footnote: Imprecise ICD-10 codes (R99,R96.0,R57.9,R40.2,R09.2,I46.9,I99,I95.9,J96.0,J96.9,P28.5) excluded. N = 386 486.

When the UCD was a neoplasm, consistencies reached 93.7% and the MD of last hospital stay was more often similar to UCD (54.9%), much higher than for any other cause of death (Figure 2). Consistencies were around 83% for the other categories of UCD, similarities varying from 17.9% for neuro-sensorial diseases to 37.7% for digestive diseases.

By definition, for affections due to external causes, MD should belong to ICD-10 Chapter XIX [13] and UCD should belong to chapter XX [11]. They can therefore never be similar, but such cases were detected as acceptable sequences by our algorithm.

### Independence vs. consistency

Considering in-hospital deaths, after exclusion of non-informative cases, independence represented 8.7% of cases (Table 2). It was higher for deaths occurring after 65 years of age, longer last hospital stays (ptrend < .0001), and UCD others than neoplasms. Adjusted results were similar and revealed a trend of increasing independence when the number of hospital stays during the last year of life increased.

**Table 2:** Frequency of independence between main diagnosis (MD) and underlying cause of death (UCD) according to age, gender, discharge-death time interval, length of stay, number of hospital stays and category of UCD and relative risks (univariate and multivariate analysis)

	**In-hospital deaths**					**Out-of-hospital deaths**			
	**n**	**Independence**	**RR**	**RRa**		**n**	**Independence**	**RR**	**RRa**
	289 904					81 688			
**Age (years)**									
**1-14**	1 010	5.2%	0.58*	0.57*		154	11.7%	0.59*	0.52*
**15-34**	3 576	4.8%	0.52*	0.55*		917	24.9%	1.26*	0.87*
**35-64**	71 556	7.1%	0.78*	0.87*		14 562	21.6%	1.09*	1.10*
**65-84**	143 413	9.1%	1	1		36 314	19.8%	1	1
**>84**	70 349	9.7%	1.07*	0.97*		29 741	22.5%	1.14*	0.96*
***Ptrend***			*<.0001*	*<.0001*				*0.0961*	*<.0001*
**Gender**									
**Male**	158 793	8.8%	1	1		39 582	20.8%	1	1
**Female**	131 111	8.6%	0.98	0.93*		42 106	21.5%	1.03*	0.98
**Discharge-death time interval (months)**									
**[0-1]**						34 678	15.1%	1	1
**[1-3]**						17 548	20.6%	1.36*	1.31*
**[3-6]**						17 691	26.8%	1.77*	1.53*
**[6-12]**						11 771	31.0%	2.05*	1.62*
***Ptrend***								*<.0001*	*<.0001*
**Length of last stay (days)**									
**[0-1]**	22 622	3.9%	0.39*	0.33*		8 677	23.5%	1.21*	1.19*
[1-3]	73 259	6.8%	0.67*	0.60*		15 722	23.2%	1.19*	1.09*
[4-9]	73 751	9.2%	0.92*	0.87*		20 983	22.0%	1.13*	1.06*
[10-29]	89 435	10.1%	1	1		30 028	19.4%	1	
**>30**	30 837	11.4%	1.13*	1.16*		6 278	17.9%	0.92*	0.98
***Ptrend***			*<.0001*	*<.0001*				*<.0001*	*<.0001*
**Number of hospital stays within the year before death**									
**1**	96 470	8.6%	0.96*	0.90*		31 150	23.5%	1.30*	1.08*
**2-3**	105 464	9.0%	1	1		30 037	23.2%	1	1
**4-5**	46 523	8.8%	0.98	1.08*		11 522	22.0%	0.81*	0.96
**>6**	41 447	8.0%	0.89*	1.12*		8 979	19.4%	0.72*	1.03
***Ptrend***			*<.0001*	*<.0001*				*<.0001*	*<.0001*
**Category of UCD**									
**Neoplasms**	122 824	5.4%	1	1		29 807	9.8%	1	1
**Neuro-sensorial disease**	9 456	11.3%	2.11*	2.33*		5 900	25.0%	2.55*	2.33*
**Circulatory disease**	71 702	10.1%	1.88*	2.21*		21 872	27.9%	2.84*	2.71*
**Respiratory disease**	19 894	9.3%	1.74*	1.93*		4 067	23.2%	2.36*	2.28*
**Digestive disease**	19 796	11.7%	2.18*	2.51*		2 470	29.1%	2.97*	2.85*
**External causes**	13 126	9.4%	1.74*	2.32*		5 453	30.7%	3.12*	2.71*
**Other**	33 106	14.9%	2.78*	3.15*		12 119	28.2%	2.88*	2.68*

non-informative UCD excluded.

RRa: adjusted for age. sex. length of stay. number of stays during last year of life. and category of UCD.

*p < 0.05.

Considering out-of-hospital deaths, independence represented 21.1% of cases. The proportion was especially high for deaths in the 15–34 years class, or for death with an external cause. It was positively associated with the discharge-death time interval (ptrend < .0001) and negatively with length of stay (ptrend < .0001) and number of hospitalizations in the last year of life (ptrend < .0001). After adjustment, the strength of these associations weakened, but the associations with discharge-death time interval and with category of UCD other than neoplasms remained noticeable. Age class 15–34 years was no longer associated with an increased risk of independence.

## Discussion

### Principal findings

We proposed an automatic method of comparison of the main diagnosis (MD) of the last hospital stay to the underlying cause of death (UCD) relying on Iris software, in order to determine their consistency or independence. This method proved able to analyze automatically 91.7% of the 421,460 submitted deaths having occurred in France in 2008–2009 within one year from last discharge. The main reasons for rejects were MD not accepted as valid causes of death and iatrogenicity.

In most cases, MD of last hospital stay and UCD were consistent, or in other words, referred to a same train of events leading to death: 88.8% of in-hospital death and 72.9% of deaths occurring after discharge.

The distribution of consistency and independence according to socio-demographic and medico-administrative variables gave expectable results: independence was more frequent in elder patients, likely because they suffer from multi-pathologies, or as the discharge-death time interval grew (8.5% of in-hospital deaths, 14.3% when death occurred within one month after discharge and 27.7% within 6 to 12 months), or for non-neoplasms UCDs, which had already been noted in former studies [5,6,8].

A long last stay or numerous hospitalizations in last year of life were associated with higher independence for people dying in hospital but with lower independence for people dying out-of-hospital, which may seem a paradox. However, independence was still lower for patients deceased in hospital after a very long stay (11.4%) than for patients deceased after discharge of a very short stay (17.9%). Possibly, these are markers of severe and complex medical situations that more often involve multi-pathologies. One hypothesis behind these results would be that complexity explains the result for in-hospital deaths, but in the same time, severity is associated with a greater probability for the physician certifying the death to recall the hospital main diagnosis for out-of-hospital deaths.

Besides, this study has shown that MD should not be used as a proxy of the UCD, even for patients deceased in hospital, since MD and UCD are similar in only 40% of in-hospital deaths. This result accounts for the difference of definition and coding context of MD and UCD.

### Results in relation to other studies

The levels of consistency found in France are similar to those previously measured in Sweden: 89% for in-hospital deaths in both countries and 71% in France vs. 68% in Sweden for out-of-hospital deaths [7]. In the Swedish study, hospital case summaries for some hospital deaths were investigated showing that inconsistency between MD and UCD was often due to certification errors. Among non-consistent cases, our algorithm was designed to distinguish independency and non-informative death certificates. However, only a look back on a series of medical records will assess the rate of coding errors on MD or certification errors on UCD leading to misclassification.

### Strengths

We have proposed a formal definition of the concept of independence between the main condition treated during a hospital stay and a subsequent death.

Using Iris Software, we have designed a language-independent method of comparison of ICD-10 codes of MD and UCD, which is based on international standards. The use of international procedures and tools guarantees that the method can be reproduced by any country. This widely automated method makes the comparison feasible on very large national datasets.

### Limits

The appraisal of independence could likely be improved. Generally speaking, independence is probably under-estimated because the MMDS knowledge table through which the causal relation are judged was designed to appraise causality between two causes, knowing that a medical doctor had declared them linked. They therefore accept "possible causality". A way to limit this bias would be to build a stricter table, aiming at only capturing "probable causality".

Alternatively, in some cases, independence is likely to be over-estimated. An example is the recording of two different primary neoplasms as MD and UCD, resulting in an "independent" label, whereas one of the codes is probably erroneous. Indeed, in some frequent cases, both codes most likely refer to the same pathology (eg: malignant neoplasm of colon (C18) and malignant neoplasm of rectum (C20)). More generally, users' feedback might bring a few improvements of the algorithm and reduce these issues.

Another source of independence over-estimation lies in considering only the hospitalization main diagnosis. For patients suffering from multiple pathologies, this may lead to the labeling "independent", whereas a pathology similar or causally related to the UCD was in fact taken care of during the hospital stay. Comparing all the conditions mentioned in the hospital discharge abstract to the UCD would resolve this; it would need the development of a more complex algorithm.

### Future research on hospital quality of care assessment

We believe that the concept of independence between MD and UCD, along with the practical method of appraisal exposed herein, could be useful for the construction of post-hospital mortality indicators. Mortality is currently used in several countries to compare hospitals quality of care, although this has been criticized [19-21]. For example, hospital standardized mortality ratios (HSMRs) are used in an increasing number of countries including England, Canada and the United States [22-24]. Relying on hospital administrative data only, these indicators give an overall measure of in-hospital mortality, adjusted for available case mix factors. Nevertheless, factors such as length-of-stay and transfer or discharge patterns, which vary between hospitals, affect in-patient death rates [25-28]. Therefore, taking into account the deaths occurring out of hospital and using time-based indicators such as total 30-days from admission mortality is necessary and has a significant impact on HSMRs [23]. On the other hand, mortality after discharge is linked to several other factors and may not reflect actual hospital performance [29-33]. Indeed, our results confirm that the longer the time after discharge, the higher the probability for an independent cause of death to occur. The exclusion of deaths independent of the MD from the deaths allocated to the hospital might thus improve the accuracy of potential mortality-based quality-of-care indicators.

The method exposed in this paper is general and potentially applies to all MD/UCD configurations. However, for some specific issues, further developments are necessary. This is the case for iatrogenicity which is systematically rejected by Iris. Although these cases are rare (3.2%) and likely do not influence much HSMR estimates, they are informative for quality of care evaluations, and should therefore be studied following a different methodology.

## Conclusions

The method presented in this paper permits us to obtain more structured and exploitable information from large hospital and mortality datasets. It is still to be improved, but the distribution of the relation obtained is mainly compatible with what would be expected.

Causes of death could improve hospital mortality indicators built for evaluating and improving hospital quality and future research on post-hospital mortality indicators should take the notion of independence between hospital diagnoses and underlying cause of death into account.

More generally, this method has the potential of being developed and used for other diagnoses comparisons across time periods or databases.

## Competing interests

The authors declare that they have no competing interests.

## Authors’ contributions

ALV, GR, GP, LAJ and EJ conceived the study idea and design. ALV, GR and EJ were responsible for data linkage. ALV, GR and GP were responsible for elaborating and running the algorithm, and analysing the data. ALV drafted the manuscript. All authors participated in interpreting the data and revising the manuscript. All authors read and approved the final manuscript.

## Pre-publication history

The pre-publication history for this paper can be accessed here:

http://www.biomedcentral.com/1472-6947/14/44/prepub

## Supplementary Material

Additional file 1 **UCD/MD comparison algorithm, complementary information.** Algorithm of comparison of the main diagnosis and the underlying cause of death, figure. Click here for file

Additional file 2 Test certificates, complementary information. Click here for file

Additional file 3 The Iris software, complementary information. Click here for file
