# Supplementary material for: Automated comparison of last hospital main diagnosis and underlying cause of death ICD10 codes, France, 2008–2009
Source: BMC Med Inform Decis Mak. 2014 Jun 5;14:44. doi: 10.1186/1472-6947-14-44 (PMC4057818; doi:10.1186/1472-6947-14-44)
Supplement: Additional file 1 — UCD/MD comparison algorithm, complementary information. Algorithm of comparison of the main diagnosis and the underlying cause of death, figure. [file 1472-6947-14-44-S1.zip › additional file 1/1897347850102973_add2.pptx]

## Slide 1
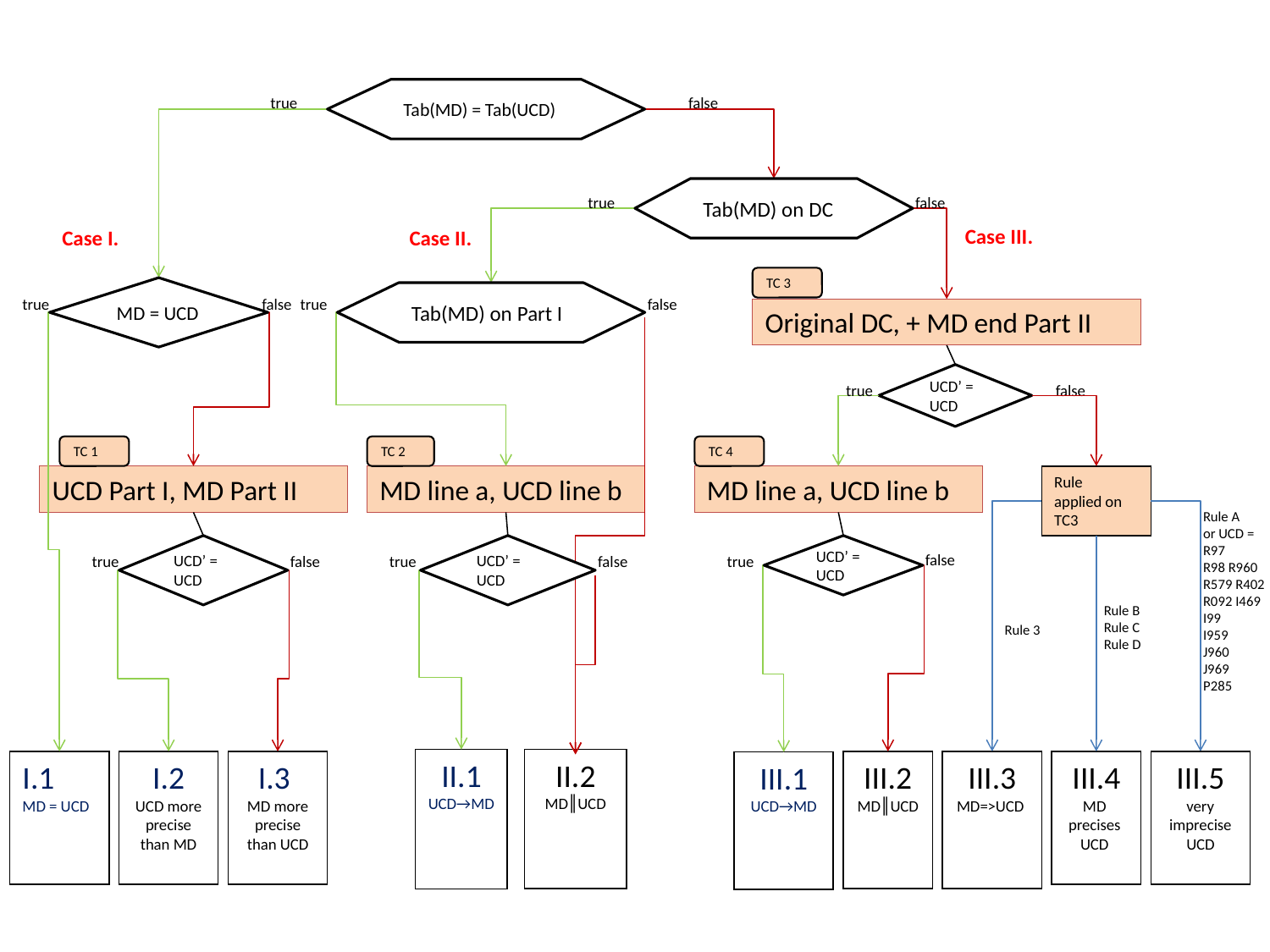

Tab(MD) = Tab(UCD)
true
false
Tab(MD) on DC
true
false
Case III.
Case I.
Case II.
TC 3
MD = UCD
Tab(MD) on Part I
true
false
true
false
Original DC, + MD end Part II
UCD’ = UCD
true
false
TC 1
TC 2
TC 4
UCD Part I, MD Part II
MD line a, UCD line b
MD line a, UCD line b
Rule
applied on TC3
Rule A
or UCD = R97
R98 R960
R579 R402 R092 I469
I99
I959
J960
J969
P285
UCD’ = UCD
UCD’ = UCD
UCD’ = UCD
false
true
false
true
false
true
Rule B
Rule C
Rule D
Rule 3
II.1
UCD→MD
19%
II.2
MD║UCD
5%
I.1
MD = UCD
15%
I.2
UCD more precise
than MD
6%
I.3
MD more precise
 than UCD
9%
III.2
MD║UCD
10%
III.3
MD=>UCD
1%
III.4
MD
precises
UCD
3%
III.5
very imprecise
UCD
3%
III.1
UCD→MD
29%
