# Supplementary material for: Automated comparison of last hospital main diagnosis and underlying cause of death ICD10 codes, France, 2008–2009
Source: BMC Med Inform Decis Mak. 2014 Jun 5;14:44. doi: 10.1186/1472-6947-14-44 (PMC4057818; doi:10.1186/1472-6947-14-44)
Supplement: Additional file 2 — Test certificates, complementary information. [file 1472-6947-14-44-S2.doc]

**Additional file 2: Test certificates**

For each concerned stay, a test certificate (TC) was constructed including only MD and UCD (TC1-2, TC4), or MD and the codes of the entire original death certificate (TC3). The location of each diagnosis on the TC is specified, according to the denominations used in the International Form of Medical Certificate of Cause of Death (Part I, line a to d, and Part II) described in ICD-10 volume 2*. Iris applies ICD-10 volume 2* rules and guidelines for mortality coding and returns automatically the corresponding underlying cause, called UCD’.

* http://apps.who.int/classifications/icd10/browse/Content/statichtml/ICD10Volume2_en_2010.pdf

**TC1:**

Concerned stays: Tab(MD)=Tab(UCD) but MD ≠UCD

Design: UCD Part I, MD Part II

Result: if UCD' = UCD, then MD is not more specific than UCD (I.2)

if UCD' = MD, then MD is more specific, Iris applies rule D (Specificity) (I.3)

**eg:** MD = C16.1 Malignant neoplasm of fundus of stomach

UCD= C16.9 Malignant neoplasm of stomach, unspecified

UCD'=MD, thus MD is more specific than UCD

**TC2:**

Concerned stays: Tab(MD) on Part I

Design: MD Line a, UCD Line b

Result: if UCD'=UCD, then UCD is an acceptable cause of MD (II.1)

if UCD'=MD, then MD and UCD have no evident causal relationship, Iris applies Rule 2 (II.2)

**eg1:** MD =I85.0 Esophageal varices with bleeding

UCD=K70.3 Alcoholic cirrhosis of liver

UCD'=UCD, thus UCD is an acceptable cause of MD

**eg2:** MD =C18.7 Malignant neoplasm of sigmoid colon

UCD=E10.2 Insulin-dependent diabetes mellitus with renal complications

UCD'=MD, thus MD and UCD are independent

**TC3:**

Concerned stays: Tab(MD) not on death certificate

Design: Original DC, MD end Part II

Result: if UCD'=MD and Rule 3 is applied, then UCD is a direct consequence of MD (III.3)

if UCD'= combination(of MD and UCD) and Rule B, C, or D is applied, then MD precises UCD (III.4)

if UCD'=MD and Rule A is applied, then UCD is an ill-defined / trivial condition (III.5)

**eg:**  MD = C18.7 Malignant neoplasm of sigmoid colon

UCD =J18.1 Lobar pneumonia , unspecified

UCD'= MD, rule 3, thus UCD is a direct consequence of MD

**TC4:**

Concerned stays: Tab(MD) not on death certificate

UCD is not a direct consequence of MD nor a less precise condition.

Design: MD Line a, UCD Line b

Result: UCD’=UCD if UCD is an acceptable cause of MD,

UCD’=MD if MD and UCD have no evident causal relationship, Iris applies Rule 2

**eg:** see examples TC2
