# Supplementary material for: Automated comparison of last hospital main diagnosis and underlying cause of death ICD10 codes, France, 2008–2009
Source: BMC Med Inform Decis Mak. 2014 Jun 5;14:44. doi: 10.1186/1472-6947-14-44 (PMC4057818; doi:10.1186/1472-6947-14-44)
Supplement: Additional file 3 — The Iris software, complementary information. [file 1472-6947-14-44-S3.doc]

**Additional file 3: The Iris software**

Available from: http://www.cepidc.inserm.fr/inserm/html/IRIS/iris_project.htm

Iris is an interactive coding system dedicated to the coding of causes of death and to the selection of the underlying causes of death.

The aim of Iris is twofold.

• To propose a language independent system. Language aspects are separated from the software and stored into tables in a database.

• To improve international comparability. Iris is based on the international form of death certificate provided by WHO. The causes of death coding follows WHO ICD-10 rules and guidelines. On the other hand, the selection of the underlying cause of death is entirely based on components of the MMDS software developed by the US National Centre for Health Statistics (NCHS).

Iris allows to process automatically a large number of cases.

Iris has been designed and developed on the basis of a collaboration of several countries including France, Germany, Hungary, Italy, Sweden and United States.  It is currently used and updated by a steering group of 10 countries, including Germany, France and Sweden (2012).

Given the objectives of the Iris project, it is important to keep Iris an international product thus Iris is a free piece of software but is not open source: Iris evolutions are studied by a user group opened to each country using Iris.

An Iris Institute has been created in September 2012. It is hosted by the DIMDI depending on the German Ministry of Health (www.iris-institute.org).
