# Supplementary material for: Automated comparison of last hospital main diagnosis and underlying cause of death ICD10 codes, France, 2008–2009
Source: BMC Med Inform Decis Mak. 2014 Jun 5;14:44. doi: 10.1186/1472-6947-14-44 (PMC4057818; doi:10.1186/1472-6947-14-44)
Supplement: Additional file 1 — UCD/MD comparison algorithm, complementary information. Algorithm of comparison of the main diagnosis and the underlying cause of death, figure. [file 1472-6947-14-44-S1.zip › additional file 1/1897347850102973_add1.doc]

**Additional file 1: UCD/MD comparison algorithm**

In a first step, cases which could not be automatically analyzed were excluded. This concerns sequences including a notion of iatrogenicity (N=13 476), stays with a chapter XXI code as main diagnosis and no related diagnosis (N=12 371), stays with an asterisk code as main diagnosis (chapter 3.1.3. of ICD-10 volume 2.)(N=4 061).

In a second step, MD, UCD and all causes present on the death certificate were translated into categories of a tabulation list for Morbi-Mortality, in order to eliminate differences that have no or little clinical relevance. The three-digit ICD-10 ‘Tabulation List for Morbidity’ (TLM) is a shortlist of 298 categories. It does not include ICD-10 External Causes chapters. The eight categories of external cause of death of ICD-10 Tabulation Lists for Mortality were added to the TLM, thus forming a tabulation list for Morbi-Mortality (TLMM) of 306 categories.

Since the ICD instructions state that the certifier's opinion should not be 'disregarded lightly', cases were analyzed depending on the presence and the location of MD on the death certificate at tabulation list level (on Part I or Part II).

The first question was: 'at TLMM level, is MD equal to UCD?'

If so (Case I.), the comparison was made at detailed ICD-10 level. The ICD-10 codes were either strictly equal (Case I.1.), unequal but nevertheless compatible - UCD being more specific than MD (Case I.2.), or MD being more specific than UCD (Case I.3.) - or different (although in the same TLMM category) (Case I. else.) [TC1].

If MD was dissimilar to UCD at TLMM level, the next question was: “is MD present anywhere on the death certificate at TLMM level? “

If so, it was assumed that the certifier had reported the condition also reported as MD in the correct part of the death certificate. Thus, if MD was reported in Part II, it was assumed that MD was not involved directly in the death process, but that it rather was a contributing independent condition (case II.2).

Alternatively, if, at TLMM level, MD was present in Part I of the death certificate (and still unequal to UCD), we used test certificates to check whether UCD could have caused MD (case II.1.) or not (case II.2) [TC2].

If at TLMM level MD was not coded anywhere on the death certificate, we wondered whether the condition reported during hospital stay could have been wrongly omitted on the death certificate [TC3]. As a matter of fact, UCD might be obviously a direct consequence of MD in the death process (case III.3), or a less precise condition (case III.4 or III.5).

If MD was omitted from the death certificate for no obvious reason, the last series of test certificates was ran in order to know whether UCD could have caused MD (case III.1.) or not (case III.2) [TC4].

Correspondence between detailed 10 categories and summarized 4 categories:

Similarity = I.1 + I.2 + I.3 + III.3

Acceptable sequence = II.1 + III.1

Independence = II.2 + III.2

Non-informative death certificate = III.4 + III.5

The comparison algorithm is diagrammed in Additional file 1bis.

**Additional file 1bis : algorithm of comparison of the main diagnosis and the underlying cause of death**

**Legend:**

MD: main diagnosis

UCD: underlying cause of death

Tab: tabulation list

DC: death certificate

TC: test certificate

A→ B : A is an acceptable cause of B

A => B : B is a direct consequence of A

A || B : A and B are independent

***(see JPEG file:* Additional file 1bis*)***
